# Analyzing the diffusion of feminist discourses on Chinese social media: A case study of the 2022 Tangshan restaurant attack

**DOI:** 10.1371/journal.pone.0308870

**Published:** 2024-08-23

**Authors:** Gege Fang, Zitong Hong, Guanting Chen, Jingwen Wang

**Affiliations:** 1 School of Digital Media and Design Arts, Beijing University of Posts and Telecommunications, Beijing, China; 2 School of Culture and Communication, The University of Melbourne, Parkville, VIC, Australia; 3 School of Psychological and Cognitive Sciences, Peking University, Beijing, China; 4 School of Television, Communication University of China, Beijing, China; University of Albany, State University of New York, UNITED STATES OF AMERICA

## Abstract

Network platforms have ushered in a novel propagation model for feminist discourses. The emergence of oriental feminism in society has led to gender-based public opinions surrounding public events becoming a trending topic on Chinese social media. This study uses the 2022 Tangshan restaurant attack as a case study, an incident that sparked widespread discussions across China in 2022. The research gathered 366,602 network communication nodes within a week and examined the communication networks of three types of content nodes (information, opinion, and appeasement) using the complex network modeling method. The findings revealed that all three types of information communication networks exhibit an apparent scale-free characteristic, and the "key minority" of nodes significantly affects information communication. Information-type and appeasement-type Weibo display notable similarities in the quantity and degree distribution of nodes within the communication networks and in the information decay rate. Moreover, authoritative information issuers have become the primary catalyst for information propagation. Conversely, opinion-type Weibo has the widest communication network diameter and features a high degree of participation, multilevel propagation, and a slow decay rate. This indicates that the interaction between opinion leaders and netizens has enhanced the depth and breadth of information diffusion for opinion-type Weibo.

## Introduction

On June 10, 2022, an intoxicated individual attempted to harass a woman who was enjoying a meal with three of her friends at a barbecue restaurant in Tangshan, China. When met with opposition, his actions escalated into a group assault, involving his companions, against the four women. This incident was captured on video and shared on social media, sparking heated discussions on Chinese platforms. The week following the incident, the tags related to "the 2022 Tangshan restaurant attack" on Sina Weibo totaled 108, with 54 garnering over 100 million views per topic. *The People’s Daily*, *China Women’s News*, *CCTV News*, and 15 other official media outlets and government agencies at various levels participated in the online discussions and publicized the case. The roles played by Chinese mainstream media and governmental agencies in social movements differ from those in the West; before, journalists from China’s official media rarely participated in discussing such issues [1]. In the feminist online movement, official media and agencies act as participants rather than initially serving as mediators [2]. Due to this case involving women’s safety, the Tangshan restaurant attack quickly escalated into a public discussion about gender opposition and controversial hate speech. Tens of thousands of Weibo users have publicly criticized the violent event and promoted extreme gender stereotypes of men as “irritable,” “violent,” and “trampling on women.” In contrast, some netizens have criticized the behavior of women going out late at night. This image description has also appeared in the previous "Didi ride-hailing driver rapes woman passenger to death" case that occurred in 2018. In just four years, China’s social media platforms have witnessed several such public discussions on gender issues, sparking the negative phenomenon of "hate speech" and "gender opposition" in the same way, which also gives a different meaning to feminist discourse.

This case has garnered significant attention on Chinese social media. The last two decades have seen a significant increase in women’s voices on social media to address gender issues in China, with feminists using social media as a primary venue for online activism [3]. Although these voices provide more information and perspectives [4], they have also intensified online campaigns on gender issues. Unlike in Western democracies, social media in China is subject to state censorship, but the rapid expansion of data makes much information unregulated [5]. Initially, in public safety incidents involving women, the discourse often shifts from strict adherence to the facts to a generalized gender-based attack, such as "men berating women" [6], which in turn sparks a collective phenomenon of gender opposition. Since its introduction to China, feminism has seen a burgeoning growth in the digital sphere. Highly influential academics and celebrities have engaged in online debates about "the 2022 Tangshan restaurant assault," strengthening the social and psychological connections between various groups. Moreover, netizens, key opinion leaders, and experts interacting at different points in these online gender debates play varying roles in shaping the speed and effect of opinion dissemination [7]. The online discourse surrounding the incident and its paths is worthy of study.

This study introduces the complex network analysis modeling, which plays a vital role in analyzing the information diffusion in social media through a contentious public issue. The method of complex network analysis can capture digital traces of human communication behavior, using massive data, text mining, and data analysis of notable computational features to describe various topologies to reproduce multiple dynamic behavior characteristics. Analyzing the information diffusion of public opinion through network nodes could help understand the public opinion diffusion routes of hate speech related to gender issues and how the involvement of different groups in gender discussions could affect the communication effect. This could have significant implications for predicting and controlling the diffusion of hate speech and group opposition under gender issues to prevent the emergence of more radical movements. This empirical study of complex network modeling has important implications for network advocates and activists on social media platforms in China.

## Literature review

### The rise of feminist discourses in social media

In the late 1990s, the effects of globalization, digitalization, and neoliberalism led to a surge in the visibility of feminist events, with group communication in the digital space rapidly expanding its influence [8, 9]. Neoliberalism has focused on the balance and imbalance of social relations in contemporary society. Inspired by this, feminists began emphasizing gender equality and liberal ideologies [10]. In light of historical developments, the structure of feminist discourse cannot be defined simply in neutral "individual" terms. Instead, it must be considered in the context of the specific constitution concealed beneath gender power dynamics, such as male-dominated politics, power and social economy, unequal gender relations, and sexual assault and violence [11–13]. In the early 21st century, the swift advancement of digital network platforms offered technical support for establishing gender order [14], introducing a new dissemination pattern for feminist discourses [15]. As blogs and social media became the primary fertile ground for feminist campaigns [16], the long-standing "aphasic" state of women changed [17].

In China, Weibo serves as a vital digital space for internet users to access and disseminate information [18] and as one of the primary social media platforms where public issues are debated. These social media platforms connect various supporters and offer immense potential for diverse modes of online expression [19]. They create a more segmented pseudo-environment within the broader discussion environment, acting as a "petri dish" for the discourse structure of gender opposition. This continually strengthens different groups within this specific pseudo-environment [20]. The spread of feminist discourse via social media has been rapid, from the United States to Asia, Europe, Africa, and Latin America [13]. Victims, whistleblowers, policy enforcers, and digital activists have come together unprecedentedly, paying unparalleled attention to relevant events. These significant opportunities offered by social media have fostered the growth and flourishing of feminist discourse [21].

In the early 1980s, Chinese feminists were primarily educated urban women [22] who had strong ties with the country and the academic community. They were working toward establishing non-governmental organizations (NGOs) and networks and advocating for legislation against domestic violence [23]. With the advent of feminist discourse on the internet, many individuals have begun to identify as feminists and embrace feminist ideologies and values. Regardless of their socioeconomic status, these individuals have started to protest against harassment and sexual assault by sharing and discussing such incidents. The participants in these feminist movements are using the internet to challenge patriarchal and male-dominated societal values [13], encouraging more women to reject sexual violence and assault. Early feminist discourse and gender issues were confined to women’s studies in the academic field, or a few intellectuals and social elites [3]. In the early 2000s, feminism was no longer a mode of action within specific organizations but became a digital social movement in which online citizens participated. The main body of action was mainstream women discussing gender equality on non-governmental websites or online communities and a small number of LGBTQ voices [3]. In the past decade, online discussion of gender issues has attracted people from different backgrounds [24]. At the same time, it also touches on the participation of official media and organizations. The official media discourse mode mainly focuses on gender equality, women’s rights, and the protection of women’s interests (such as marriage, childbearing, vaccines, etc.). Discussions from the private sector, on the other hand, prefer to investigate impressive social and public events.

As feminist discourses continue to gain exposure, the diffusion patterns of their content start to form [25], and the gender power structure on digital platforms begins to shift. First, in the digital space, the growing influence of confident female opinion leaders means more women are moving from the margins to the center. The permeation of feminist trends into China is not a linear process, but one that coincides with the country’s economic development and social structure changes. In this context, numerous opinion leaders have emerged to steer the female discourse structure, specifically, individuals who have influenced and shaped personal cognition, attitudes, and behavior [26]. Further investigation reveals that these feminist opinion leaders have played a crucial role in advancing discussions on gender issues, stimulating empathetic emotional responses, and promoting mutual protection and assistance among women [27]. This point is further supported by the research of Ma and Hu [28]. As the number of female opinion leaders and group feminist events rises, the long-standing male dominance is challenged by feminist discourses, prompting some men to reassess their paternal roles.

Second, existing research suggests that the digital space, a significant platform for feminist movements, is highly intricate [9, 29] and paradoxical [21, 30]. The digital network has created a stage for the public to express various perspectives, leading to complex situations in cyberspace rather than simplified ones. In the monistic public sphere, as defined by Habermas—where women (categorized as the inferior group and denied participation in public issue discussions), working-class men, and certain radicals are excluded—the inferior group lacks a separate space to deliberate its objectives [31]. However, feminists have introduced topics such as "the inherent danger to women," "gender bias," "sexual harassment," and "domestic abuse" into the public domain, breaking the monistic boundaries of the traditional public domain and reshaping a digital public domain [32, 33]. Online debates on gender issues have given rise to intricate rhetorical battles of factional discourse. The internet is awash with conflicting voices, as antifeminism and post-feminism disrupt feminist narratives through nuanced storytelling [9]. Post-feminists are considered a distinct group that is highly responsive to neoliberalism, embodying the interconnected disparities of gender, race, and class in various forms. As Linabary et al.’s study illustrates, post-feminists are simultaneously feminist and antifeminist, symbolizing liberation and oppression, and posing a threat to social reform. On Chinese social media, those actively engaged in gender issues are labeled by radicals with various tags, such as "female fist," "Chinese pastoral feminism" (a term derived from the phrase “Chinese pastoral dog”), and "shivering cold," among others [8]. Given China’s lengthy history of a patriarchal social structure, the evolution of Chinese feminism faces a particularly complex public opinion landscape.

Third, the extensive use of digital media is transforming and reshaping feminism in the 21st Century, enabling it to become increasingly radical. For example, as hashtags on social media platforms evolve into a mechanism for online activism [9], any subject can quickly gain attention by accumulating tags, thereby swiftly entering the public eye [34]. Because feminism-related issues are highlighted and rapidly amplified on social media, they incite the participation of radical feminists and post-feminists, leading to vigorous online debates. Although feminist thought was introduced to China relatively late, it now exists in a stage marked by complex shifts in cultural characteristics [8], where diverse groups coexist in cyberspace amidst a plethora of gender issues, such as radical feminism, antifeminism, and misogyny [29]. The emergence of radical feminism may result in structural changes and more heated debates among differing perspectives, giving rise to gender-based online hate speech.

### Hate speech and group opposition under gender issues

Hate speech has a wide range of meanings in terms of concept and consequences. Although "hate speech" is often used as a term in the research of public opinion in the international community, there is no single definition for this explanation. Still, it is widely understood as the speech of specific individuals or groups attacking or mocking others according to the typical characteristics of external groups or individuals [35, 36]. Hate speech is a distinct type of offensive discourse that employs abusive language to communicate a hateful ideology [37]. It is always described as a behavior that incites hatred toward an individual or a group identifiable by shared attributes, such as race, religion, and gender, through means of insult, defamation, or creating a hostile environment [38]. Nockleby [39] proposed that the semantic structure of hate speech targets any specific individual or group based on characteristics, such as race, color, ethnicity, sexual orientation, nationality, or religion. Moreover, hate speech is also seen as the use of stereotypes to categorize a group and demean an individual or an organization [37].

As female communication becomes more prevalent on social media globally, both feminist and masculinist rhetoric, as well as gender-biased discourse, have become targets of hate speech [40]. A notable characteristic of gendered online hate speech, which also includes speech containing sexually aggressive content directed at women or men [41], is gender bias. This bias can manifest as hostile sexism, gender discrimination [42], and gender stereotypes, such as the expectation that women should be gentle and men should be masculine. Prior research on gender conflict issues does not clearly state that the rapid growth of digital platforms contributes to the intensification of gender hatred and the acceleration of the dissemination of gendered online hate speech. Incidents of gender opposition will always exacerbate hate speech because it seems to be a confrontation between different social groups to fight for or defend their power and interests [43]. The protection of individual power and interests is one of the main causes of identity-based hostility, and gender opposition often occurs within groups, characterized by in-group favoritism, out-of-group programs, and distinct classifications that delineate the boundaries of prejudice and discrimination [35]. Indeed, digital communication is a crucial element influencing the formation of gender order in cyberspace.

First, algorithmic technology [44] has shaped the concurrent structure of opinion leaders’ "concentric circles" and information cocoons [45]. Over the past several decades, algorithms have matured on social media, including tasks such as sorting, filtering, searching, prioritizing, recommending, and deciding, which are considered the decision-making components of code [46]. The rise of algorithms has significantly affected pre-emptive behavior [47]. Network operators have used algorithms to push information in a targeted manner, thereby expanding the reach of content and establishing broader control over discourse [38]. The prevalence of gendered online hate speech has steadily increased [48]. The content from specific groups on social media has shown a trend toward homogenization, leading to "opinion segregation" [45]. Netizens selectively access or block content based on their interests, subtly increasing their exposure to individuals or groups with similar content. When radical interpretations of locally consistent content are escalated to a global level, it often results in group polarization among different members, potentially leading to fragmentation and segregation of the collective on a larger scale [49]. The opinion leaders within both feminist and antifeminist groups are not "decentralized," but rather "centralized," either in a local or global context [45]. Chinese researchers Cao and Zhan [50] examined public issues on Sina Weibo, including where opinion leaders were involved and the effect on public sentiment. They found that most opinion leaders have only managed to generate a small amount of discussion on topics, exhibiting a distinct power-law characteristic. Furthermore, the level of interaction among opinion leader groups is minimal, with these leaders serving as the central node, which is relatively often differentiated and integrated, in social media.

Second, hate speech in the digital space is often concealed within a vast sea of anonymous content [44]. Encryption technology has effectively transformed every user into a "shield," providing anonymity on social media platforms [51]. This form of anonymity is a complex "double-edged sword" [51]. While anonymity on social media is crucial for preserving freedom of speech, it can also have negative effects by enabling individuals to use pseudonyms [52]. This anonymity can contribute to the spread of fake news and hate speech [53]. On one side, anonymity can empower some female users to engage in online discussions without fear of backlash or criticism [54]. Conversely, it can lead to a lack of civility and the emergence of uncouth behavior in online debates [55], providing users with a sense of immunity that deviates from their behavior in the offline world [56].

Third, the anonymity provided by cyberspace can affect the group mentality of individuals, creating a new context for the "anti-silence spiral" [53], which can be referred to as a pattern of "opinion reactance." When used in online debates about gender opposition issues, an uncivilized mode of expression, as opposed to a civilized one, can heighten a user’s sense of moral indignation. Discourse lacking courtesy contradicts the spirit of debate and exacerbates animosity, leading to opposition, abuse, and ridicule among internet users with differing viewpoints. While both supportive and dissenting voices are present on social media, the dissenting speech tends to draw more engagement from internet users. In a study of public sentiment surrounding contentious issues, as analyzed through social media data, Ni et al. [57] discovered that most comments expressing a definitive stance were oppositional, particularly on platforms such as Twitter and Sina Weibo, especially in discussions involving moral and legal matters. Since internet users can typically evade any real danger to themselves by simply disconnecting from the internet, they often feel emboldened to voice opposition [58]. As societal conformity diminishes, individuals are less likely to remain silent out of fear of isolation. Instead, they may feel encouraged to challenge the majority independently [59]. This was evident in the 2014 Isla Vista shooting in California, United States, where the perpetrator sought revenge against women who had rejected him, resulting in six deaths and 13 injuries. In the aftermath, the hashtag #YesAllWomen emerged on Twitter and Facebook as a counter to #NotAllMen tweets, voicing feminist criticism and defense against gender discrimination [60]. Over 1.5 million personal messages were posted, with many users sharing experiences of violence, harassment, and threats against women [61], and condemning the misogynistic group [62]. While the state’s role in defining hate speech can be seen as a limitation on freedom of speech [63], hate speech itself poses a threat to the right to equality and exacerbates inequality [38]. Specifically, in online public discussions, the negative emotions stirred by gendered online hate speech can lead to stress-induced group behaviors, amplify gender discrimination [64], and undermine the democratic nature of online debates. Consequently, this study focuses on hate speech related to gender issues as a subject of critique.

Research into the origins and significance of gender-based hate speech is crucial for understanding the dynamics of anti-feminist groups on digital platforms and for improving the management of these groups’ communication of extreme gender ideologies. In the early stages of societal development, numerous tragedies were linked to hate speech. The content of hate speech has continually evolved in response to societal changes [38]. With the advent of cyberspace, online hate speech has transcended time and space constraints, reaching a wider audience. Its effects do not diminish over time; instead, they are likely to inflict more profound collective harm due to repeated related incidents—online hate speech results in varying degrees of negative consequences for victims and bystanders [42]. YouTube users harmed by online hate speech have reported decreased website visits [65] and participation in online discussions [66]. Additionally, victims of online hate speech often experience negative emotions such as anxiety, anger, distress, or depression [67, 68], and in extreme cases, they may instigate offline violence or exhibit suicidal tendencies [67].

Gender discrimination, exacerbated by gendered online hate speech, has a heightened negative effect on women [42]. We are witnessing a new phase in the gender conflict, characterized by a disturbing increase in hostility and violent acts aimed at women in the digital space [69]. Some antifeminists resort to violent and threatening behaviors as a method of instigating social change in the online world [64]. Despite its high visibility, the prevalent feminism is often countered by another dominant discourse pattern: "popular" misogyny, a harsh expression of antifeminism. The online propagation of misogyny has cultivated a misogynistic political and economic culture [69]. Following the widespread influence of the #MeToo movement, the 2018 "Didi carpooling event" (where an online ride-hailing driver sexually assaulted and murdered a female passenger) became a trending topic on Weibo. As women voice their opposition to such sexual assault incidents, some misogynistic groups and post-feminists blame the victims, attributing the fault to women’s "inappropriate attire." This discourse structure, which places blame on the victims and perpetuates the notion of women’s inherent guilt, is saturated with the idea of male supremacy [27].

These incidents share a similar discourse structure with the "2022 Tangshan restaurant attack" that will be examined in this paper. However, another discourse that highlights the online dispute over feminism is "toxic masculinity." This term originates from the critique of a traditional perspective that portrays masculinity and fatherhood as heroic ideals and violence as an expression of masculinity, while depicting women as weaker and in a subordinate position [63, 70]. Online platforms bring together both advocates and critics of feminism, fostering a new cross-discourse pattern [19] and amplifying the spread of gender-based hate speech. In conclusion, antifeminist discourse patterns from various contexts have led to gendered online hate speech, which is manifested through group behaviors and saturates the digital space with negative emotions.

### Complex network modeling under the cross-discipline horizon

**Fig 1:**
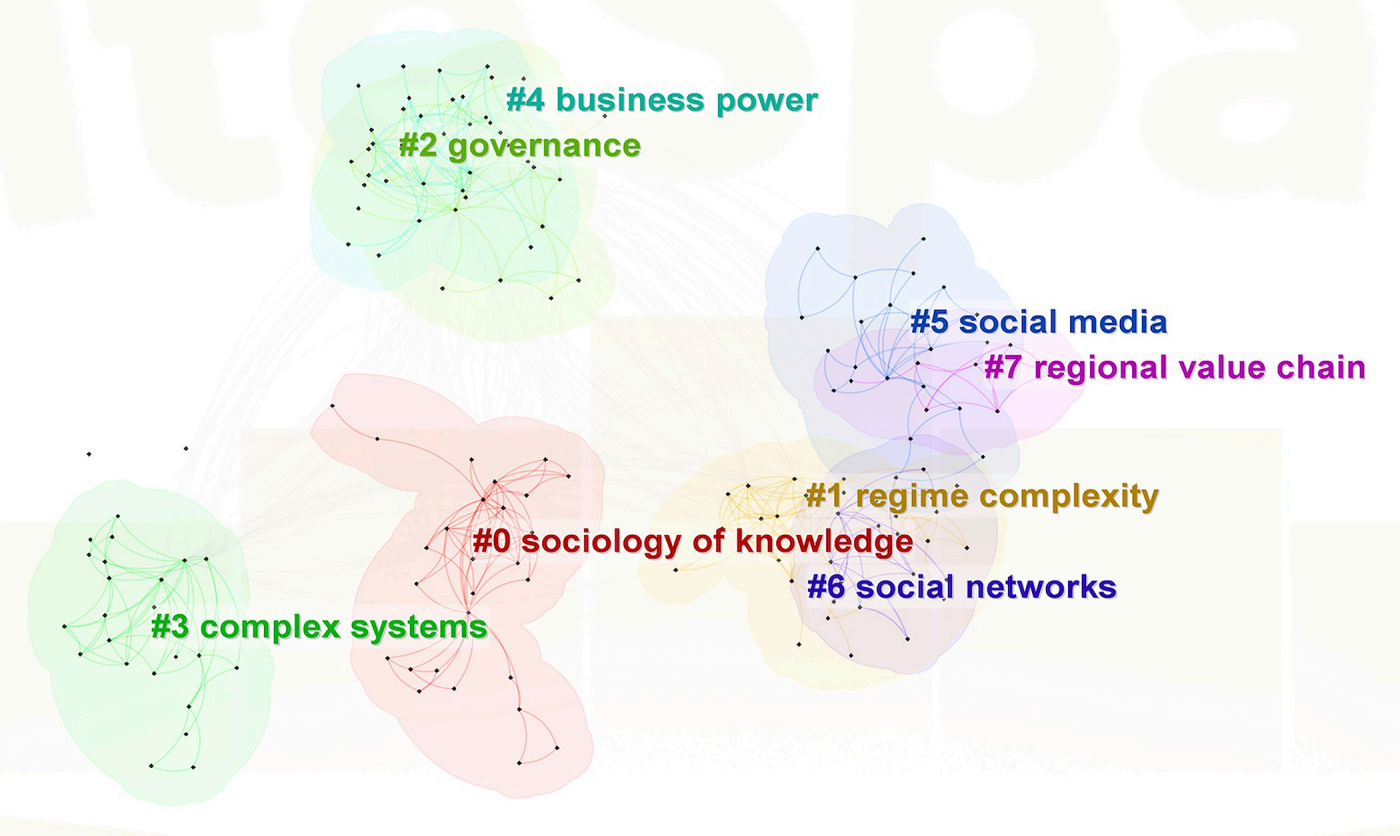
Keyword clustering network.

The emerging paradigm of computational communication research, which has seen significant application in online public opinion studies, is a distinct interdisciplinary field [71, 72]. The research paradigm originates from data and network science, focusing on complex network analysis within computational communication to study social network communication models’ qualitative and quantitative rules. This includes the study of all topologies and their properties of complex networks, their relationship with dynamic characteristics (or functions), the communication, prediction, and control of various dynamic behaviors and information on the network, and the study of the principles of the network involved in social practice [73]. The complex network allows for examining communication networks of different types of content nodes (information, opinion, and appeasement). It provides insights into how information spreads, the influence of critical nodes, and the structure of communication networks. On November 28, 2023, a study was carried out on the SSCI database using the term "complex network analysis." The search was confined to the period from 2019 to 2023, with a particular emphasis on communication, international relations, and sociology. This search yielded 234 pertinent papers. These papers were then incorporated into Citespace for a keyword clustering analysis (see Fig 1).

Fig 1 illustrates that contemporary research on complex networks primarily concentrates on interpersonal relationships in social media and real-life societies, intersecting with governmental administration, regional relationships, and media industries. Scholars in the humanities and social sciences tend to depict various network topologies and their characteristics, providing explanations through insights and case studies of social phenomena. However, they encounter challenges in modeling, which hinders their ability to conduct quantitative deductions. As a result, most of their investigations remain primarily theoretical. On the other hand, researchers in technical sciences and engineering often investigate the creation of complex network propagation dynamics models. Regrettably, their research lacks an in-depth examination of conceptual meanings and sociological mechanisms, causing these propagation dynamics models to be somewhat disconnected from social reality. Recognizing this issue, in December 2020, a research team from the School of Journalism and Communication at Tsinghua University pointed out that the limited practical value of existing models is a pressing problem that needs immediate attention in current communication dynamics research [74].

Considering this context, numerous researchers have used computational communication research to examine online public sentiment and group behaviors. In September 2021, a team from the Department of Statistics and Data Science at the Southern University of Science and Technology employed intricate network modeling and real-world Weibo observations. They discovered that the accuracy of predictions from existing propagation dynamics models is less than 2%, and the parameter prediction error is nearly a thousandfold. Subsequently, this team integrated their understanding of Weibo user behaviors into a mathematical model using technical language, ultimately achieving a significant optimization of the prediction effects. This accomplishment was featured in *Nature Human Behaviour* [75]. In March 2022, a research team from the Communication University of China conducted a propagation dynamics study on vaccine hesitancy based on authentic Twitter data. Their work was published in *Humanities & Social Sciences Communications*, a subsidiary journal of *Nature* [76].

**Fig 2:**
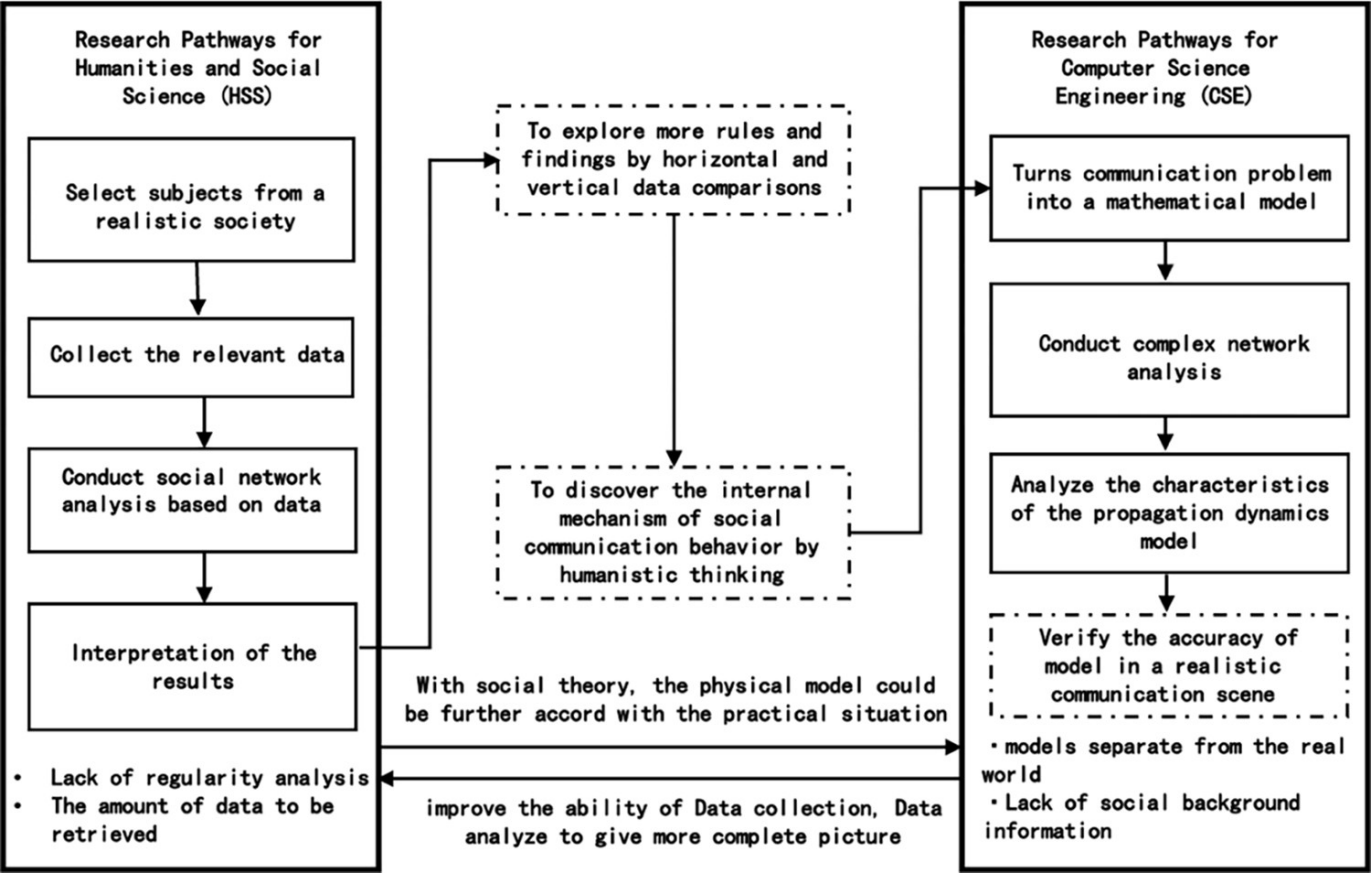
Complex network research pathways for communication studies.

Currently, scholars in the humanities and social sciences, as well as polytechnic sciences and engineering, have gradually developed unique research trajectories in the realm of computational communication research of network science, as shown in Fig 2.

In the humanities and social sciences, network science focuses primarily on illustrating the relational network of research groups, forming a distinct research trajectory. A notable characteristic of this trajectory is the emphasis on presenting and interpreting data outcomes, rather than investigating the potential existence of universal mathematical laws underlying these outcomes. The primary reason for this characteristic is the lack of sufficient data and data analysis capabilities, leading to most dissertations following this trajectory being one-time endeavors. These are concentrated in a few research areas and exhibit considerable homogeneity in research content [77]. Complex network modeling in technical sciences and engineering focuses primarily on developing propagation dynamics models, forming another distinct research trajectory. A notable characteristic of this trajectory is that the research mainly focuses on the mathematical properties of models and fails to present a comprehensive view of reality. Consequently, the research conclusions lack sufficient predictive and instructive value for social communication behavior. Compared to another standard method in big data public opinion analysis—social network analysis (the sample size usually ranges from 1 to 1000), complex network analysis can analyze more nodes and provides a broader framework for understanding the intricate and dynamic properties of various interconnected systems, which aligns with the key points we want to focus on in the diffusion of social media posts.

By employing complex network modeling, the researchers can quantitatively analyze the scale and scope of information spread, identify key influencers, and understand the dynamics of public opinion formation in digital spaces. Furthermore, this approach helps explore the relationship between the nature of the content (informational, opinionated, or appeasement) and its spread, highlighting the distinct behaviors of different types of messages in social networks. The findings on the scale-free nature of these networks and the role of authoritative information issuers underscore the model’s appropriateness for dissecting the intricate interplay of messages and their propagation paths in the context of gender discourse and public opinion on social media.

Based on the above discussion, the researchers aim to seamlessly merge the technical strengths of polytechnic sciences and engineering, particularly in complex network modeling and extensive data analysis, with the insights from humanities and social sciences. This integration is intended to bridge the gap between these two research trajectories, as depicted by the dashed boxes in Fig 2, and to establish a new research trajectory that fosters mutual enrichment between humanities and mathematics. Guided by the humanities and social sciences insights, the inherent mechanisms of realistic social communication behavior will be explored and incorporated into complex network propagation dynamics models. The effectiveness of this approach will then be evaluated in real communication settings. Among them, the scale-free characteristic of complex network analysis can reflect the situation where a minority of nodes significantly influence information dissemination. This is particularly relevant for understanding how certain posts or users become central in spreading information or opinions and how this affects the overall discourse on social media. Note that while this research trajectory has the potential to transcend disciplinary boundaries and knowledge limitations, its technical implementation may pose significant challenges, necessitating close collaboration among interdisciplinary researchers. Thus far, aside from a few comprehensive research findings that have been published in leading international journals, there have not been any substantial achievements.

Online gender-related disputes and hate speech are increasingly prevalent, hindering social agreement and democratic dialogue. This study employs complex network analysis to examine the relational data among certain users over a specific period. It seeks to uncover hidden connections within the network and the dissemination traits of various information, presenting these findings through a visualization technique. The research focuses on the "2022 Tangshan restaurant attack," a significant case that has had a substantial impact on the internet. As such, we pose the following research questions:

RQ1: What characteristics do the propagation paths of all forms of network data (e.g., information, opinion, and appeasement) exhibit in the context of gender-related matters?
RQ2: Do the type and attributes of information release accounts affect the degree of diffusion?
RQ3: Which type of information is associated with higher netizen participation than others?

## Research methodology

### Sample collection

This study focuses on the "2022 Tangshan restaurant attack," an incident on June 9, 2022, that sparked a broad spectrum of public reactions on Weibo platforms from June 10 to 17, 2022. This event has been the subject of numerous trending searches on Weibo, with hashtags such as "#More than one man assaulted a group of women at a barbecue restaurant#" and "#An attack in Tangshan#." The research team chose the hashtag "#an attack at a Tangshan restaurant#," which garnered the most page views (with 355 derivative entries and approximately 7.98 million comments) as the sample source. First, using the Python 3.7 language environment, the Requests and Scrapy modules were used to extract posts from Weibo platforms related to this event with a sharing volume exceeding 100,000.

Using a manual analysis, all posts are categorized into three distinct types: the information type, the opinion type, and the appeasement type. Information-type Weibo posts aim to objectively describe the progression of an event, devoid of any sentiment or bias. Typically, the publishers of such posts are news organizations. Opinion-type Weibo posts, primarily shared by individuals, are intended to express personal views on the discussed event and, thus, are characterized by emotionality and unique perspectives. Appeasement-type Weibo posts, disseminated by mainstream Chinese media, represent the government’s viewpoint and serve as a guide for public opinions and a pacifier for group dissent. Sorted in ascending order by the number of comments, we manually selected one post from the top three of each of the three types of posts. The criteria for manual selection were to ensure the diversity of the publishing organizations and the diversity of opinions in the comments. The three types of Weibo posts include an event’s progression shared by the news media account "@Boiling Point Video," a personal opinion against the event shared by the gender issue opinion leader "@Tian Ya Li Zhi Xing," and comments representing mainstream opinions shared by the official newspaper account of the Central Committee of the Communist Party of China "@People’s Daily" following the event. The number of shares for the three selected posts all exceeded 100,000.

In this research, we gathered data such as the identification of each user who forwarded content, the identification of each source user from whom the content was forwarded, and the time of forwarding. After eliminating missing values, we compiled 110,928 sets of event content data from Weibo user forwards, 132,837 sets of opinion content data from microblog user forwards, and 122,837 sets of appeasement content data from Weibo user forwards. After investigating the reliability of Google Translate’s translations [78], we used it to convert all sample data into English. It is important to note that the preliminary conclusion about machine translation is that researchers can use DeepL, Google Translate, or CUBBITT [79] when writing papers. However, the content translated in this study is everyday conversations from social media. Due to the universality of everyday language, the accuracy of their translations should be higher than that of translating specialized domain knowledge. These data sets formed the primary data set for our study. The raw data set we extracted is available in a data repository: https://doi.org/10.7910/DVN/D1VJJD.

### Analysis tool and research pathways

The researchers employed the complex network analysis tool, Gephi 0.9.6, to construct comprehensive networks for information communication that corresponded to the three types of information content.

**Fig 3:**
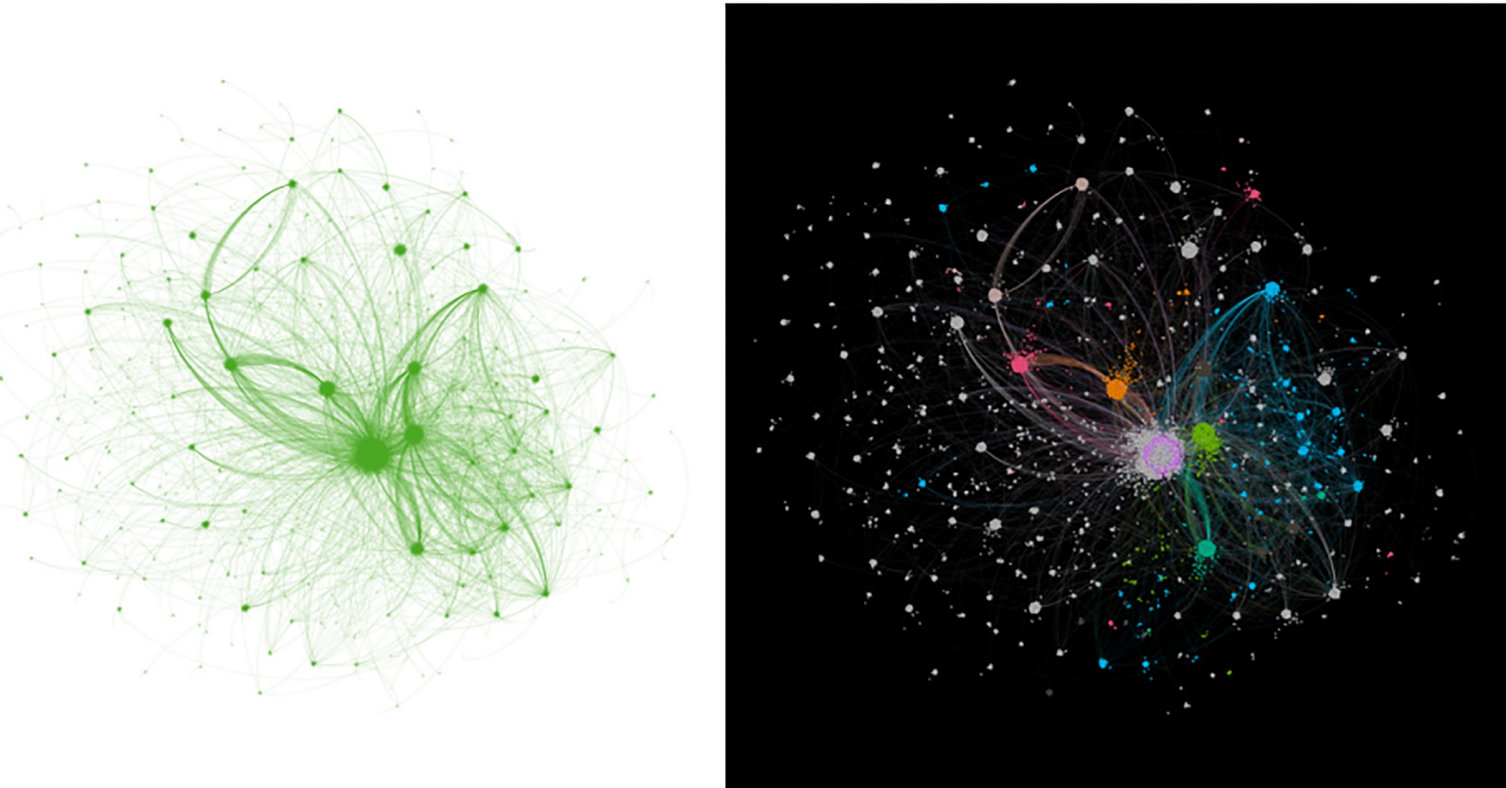
Panoramic view of the communication network for information-type Weibo content information.

**Fig 4:**
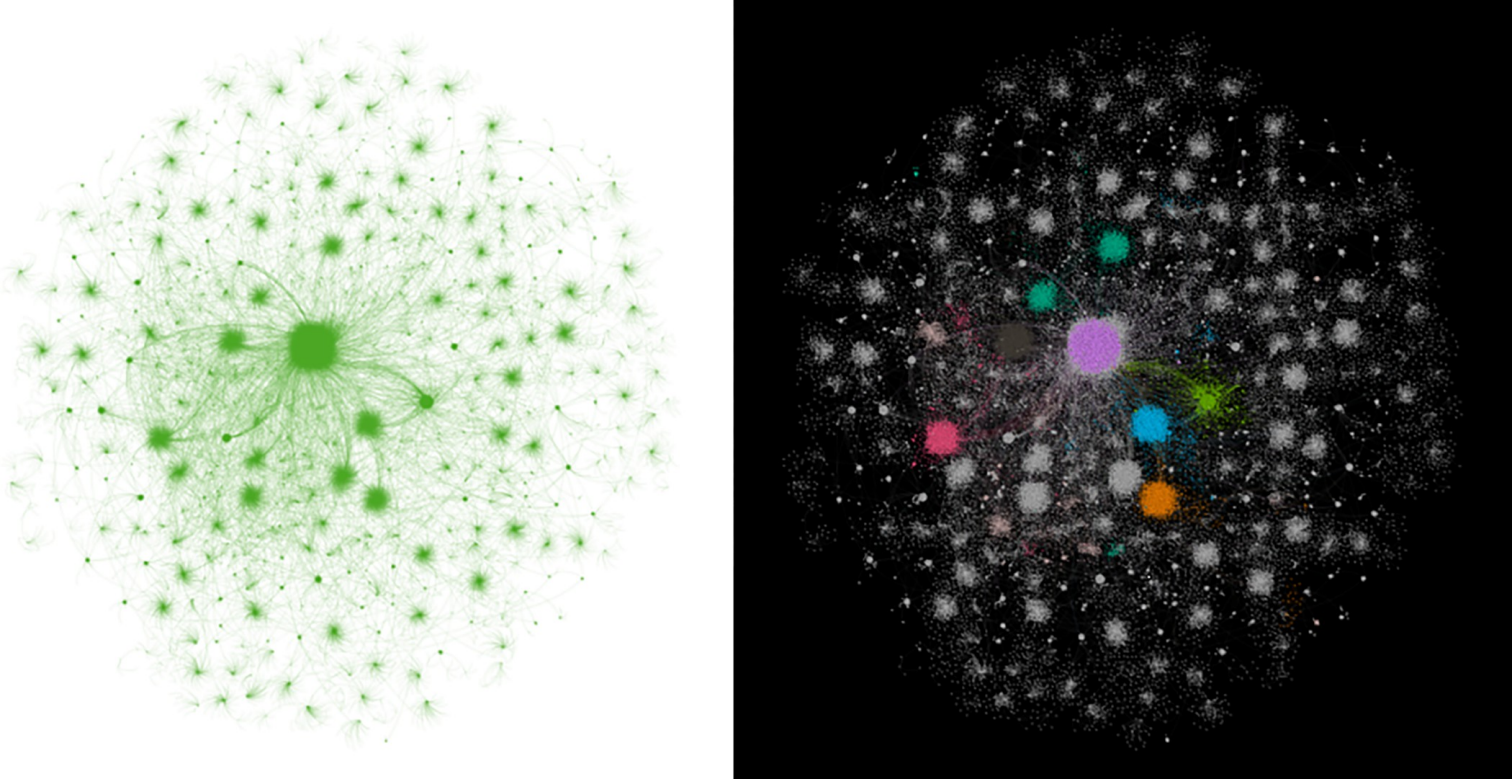
Panoramic view of the communication network for opinion-type Weibo content information.

**Fig 5:**
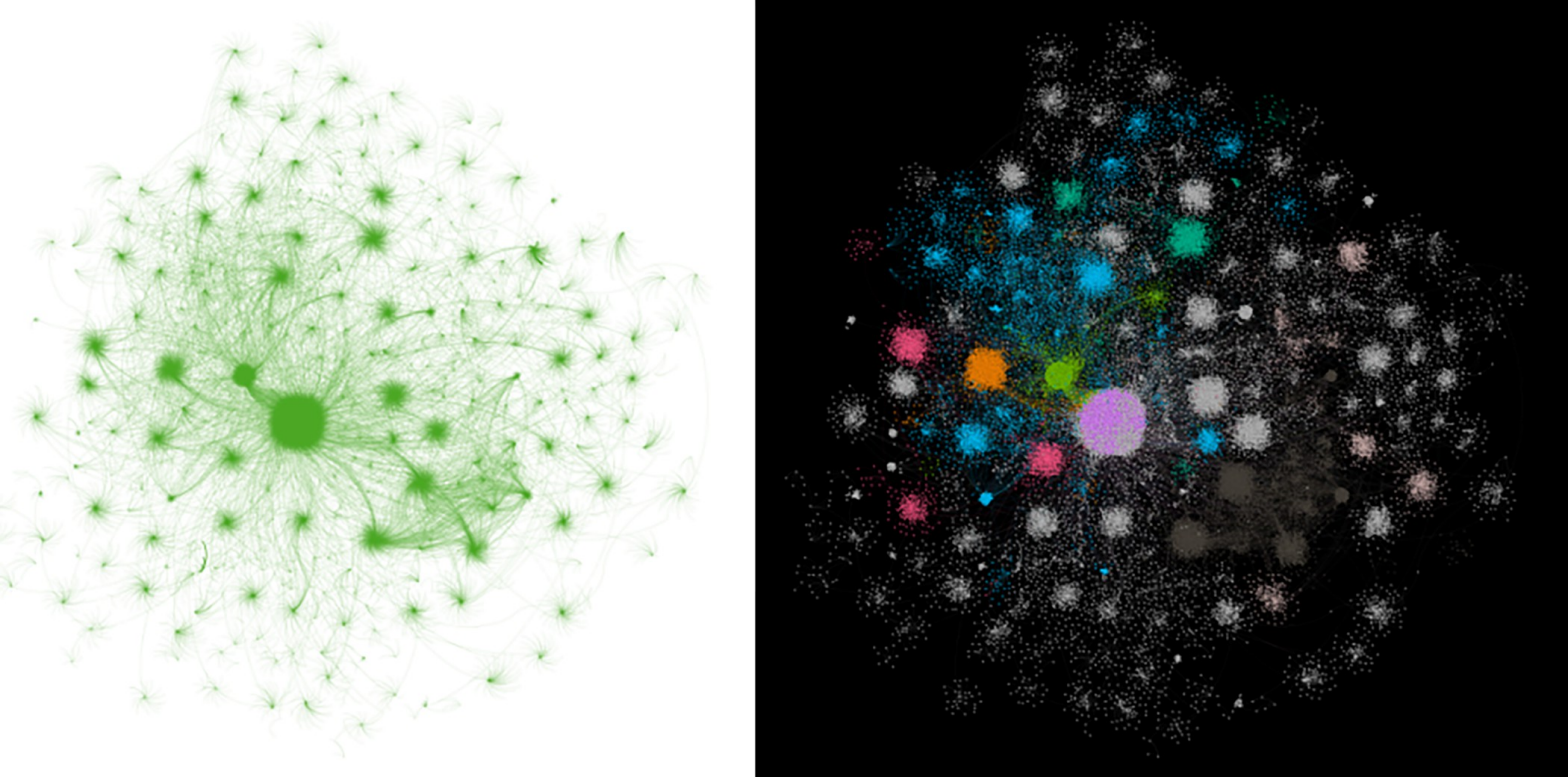
Panoramic view of the communication network for appeasement-type Weibo content information.

Based on the primary data set, The study began constructing an edge list, using user IDs as nodes and forwarding relations as edges. Note that the Weibo platforms only recorded some forwarded information, omitting data about some forwarding nodes with minimal global communication influence. This omission resulted in the network’s incomplete connectivity during its initial construction. Therefore, we enhanced the edge list by extracting Weibo content forwarded from breakpoint users. We selected the maximum connect components for filtering to ultimately form the communication network panoramas (see the diagrams on the left of Figs 3–5). The communication network for information-type content has 103,439 nodes, the network for opinion-type content has 128,600 nodes, and the network for appeasement-type content has 118,428 nodes.

As depicted in the diagrams on the left of Figs 3–5, all three communication networks present a visibly scattered point-like structure. This suggests that these networks display a pronounced scale-free characteristic, defined by a severe imbalance in the degree distribution among nodes: a small number of nodes are significantly more connected than most nodes in the network. In addition to the scale-free property, researchers often examine the small-world property of a network in complex network analysis. However, further discussion is not warranted because the connective edges in the networks constructed here are based on the forwarding relation and cannot exhibit this property. To facilitate observation, we used the built-in statistical tools in Gephi for modular processing (grouping nodes with the same or similar degree values), a standard procedure in the community detection algorithm. We treated different module groups in various ways, highlighting the groups with a large proportion of nodes (above 1%) in multiple colors, and produced the modularized panoramas, as shown in the diagrams on the right of Figs 3–5.

The diagrams on both sides of Figs 3–5 depict varying sizes of scattered nodes in distinct colors, indicating a significant difference in degree among the communication nodes within the networks. This is a crucial attribute of scale-free networks. Consequently, we investigated these communication nodes’ degree distribution and data structure. In the communication networks for the three types of Weibo content information, the depth at which information travels from the network center to the periphery seems to vary significantly. The depth of flow in the opinion-type information communication network is notably high.Additionally, the degree of nodes in the networks decreases sharply with the increase in the number of information flow layers. Visually, the depth-of-flow characteristic is primarily reflected in the difference in the number of node layers. In contrast, the decaying characteristic is reflected mainly in the trend that the size of nodes decreases significantly with the increase in the distance between them and the starting node. Therefore, we examined the flow depth along propagation paths and the decay rate.

## Research results

### Degree distribution and data structure of communication nodes

As seen in Figs 3–5, the communication nodes display a significant variation in degree throughout the network. In this research, the degree of a node is defined as the number of edges connected to that node. This variation in degree is primarily evident in the differing sizes of the dispersed nodes in the left-hand diagrams of Figs 3–5, and in the dispersed nodes’ unique colors in the same figures’ right-hand diagrams. This characteristic is another fundamental property of scale-free networks. Therefore, the researchers examined the communication nodes’ degree distribution and data structure.

**Fig 6:**
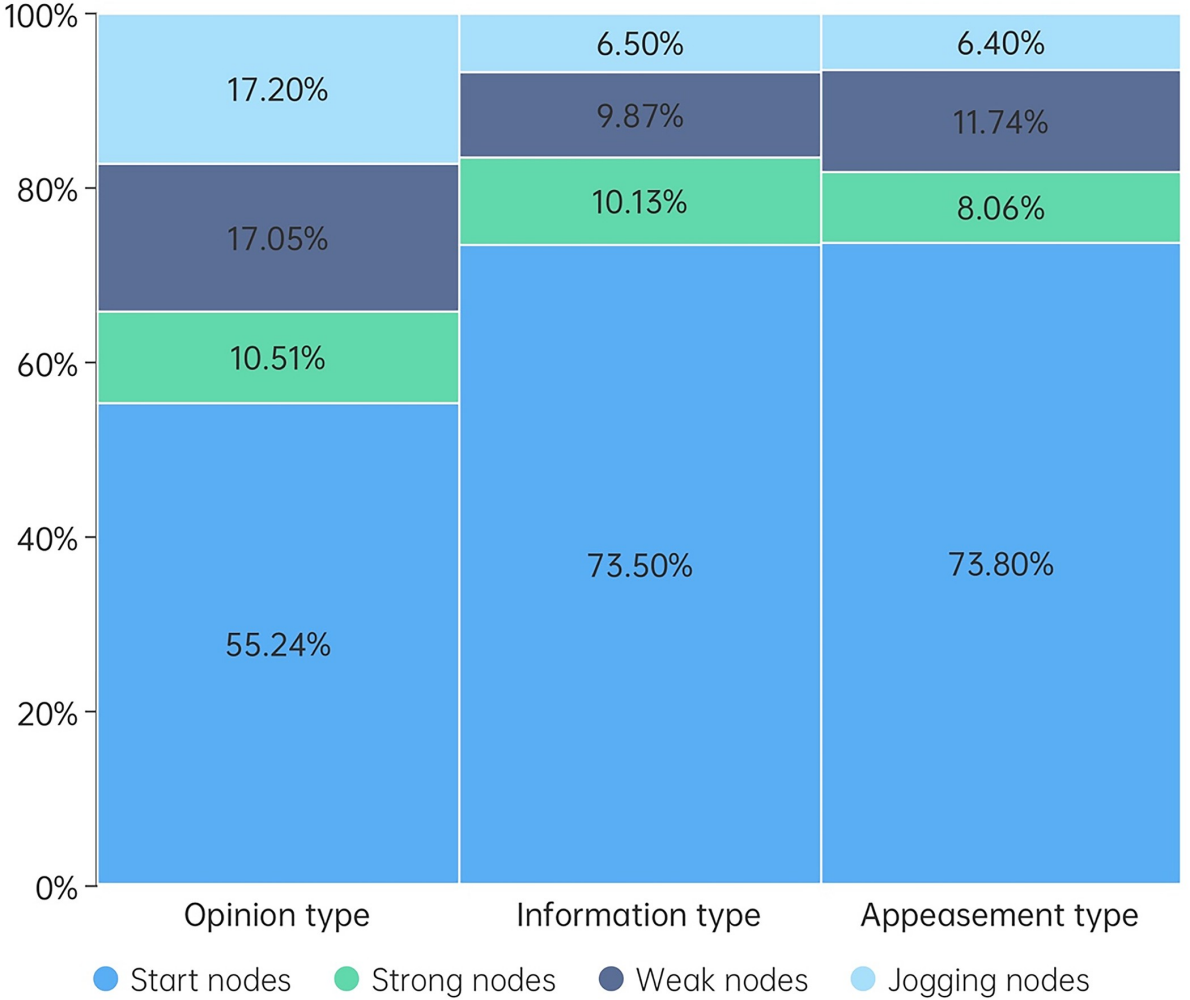
Degree distributions of nodes.

In graph theory and network analysis, "degree distribution" refers to the overall probability distribution of the number of connections each point has within a network, where "degree" signifies the number of connections a node has with other nodes. First, we determined the degrees of all nodes in three distinct types of information communication networks. Subsequently, we categorized these nodes into four types based on the degree difference: start nodes, strong nodes, weak nodes, and jogging nodes. Start nodes are the original nodes from which content is disseminated. Strong nodes are those whose degree constitutes more than 1% of the total node count. Weak nodes are those whose degree makes up between 0.1% and 1% of the total node count. Lastly, jogging nodes are those whose degree represents less than 0.1% of the total node count. The quantity distributions of all node types in the three kinds of information communication networks are given in Table 1, while their degree distributions are shown in Fig 6.

**Table 1:** Quantity distributions of nodes.

	Start nodes	Strong nodes	Weak nodes	Jogging nodes
**Opinion type**	0.0008%	0.0054%	0.058%	99.94%
**Information type**	0.001%	0.0048%	0.035%	99.96%
**Appeasement type**	0.0008%	0.0042%	0.041%	99.95%

The analysis reveals that the dissemination of three categories of Weibo content heavily relies on a "key minority" of nodes. In the case of opinion-based information, less than 0.07% of the nodes are responsible for over 82% of the communication. For event and appeasement information, approximately 0.05% of the nodes account for more than 93% of the communication. Looking at the overall degree distribution, the initiating nodes play a crucial role in the dissemination of all three types of information. While the degree of initiating nodes in the communication of opinion-based information is lower than the other two types, this deficit is compensated by weak nodes and jogging nodes. The degree proportions of strong nodes remain relatively stable, hovering around 10% across different types of information communication networks.

Note that, despite the difference in the selection of an experimental subject where news accounts under the influence of peer competition serve as the initial nodes in the transmission of information-type data. Furthermore, official organization accounts free from peer competition pressure act as the initial nodes in the transmission of appeasement-type data, both exhibit a striking similarity in the degree distribution structure of communication networks, with an average difference of less than 2%.

Lastly, we conducted a comparative analysis on the genders of participants across three categories of Weibo posts. The purpose of this gender examination was to further clarify the relevance of the chosen sample to issues related to gender. The analysis revealed that in opinion-based Weibo posts, 20.77% were from male users, while 79.23% were from female users. In information-based Weibo posts, 31.60% were from male users, and 68.40% were from female users. In appeasement-based Weibo posts, 27.09% were from male users, and 72.91% were from female users. Thus, in all three types of discussions, female users participate more, distinguishing this event from other typical online disputes. Considering that the focus of the online discourse for this event centers on "unfair treatment of women," "violence perpetrated by men," and "criminal underworld," it strikes a chord with women about their personal experiences, sparking introspection and unity. This, in turn, encourages them to form online coalitions and participate more actively in discussions or denunciations. Moreover, the researchers noted that the gender disparity is most noticeable in opinion-based Weibo posts, with female users outnumbering male users by roughly four times. This indicates that female users in this event are more actively engaged in moral judgments, emotional expressions, and value articulation.

### Depth of flow along propagation paths and rate of decay

Upon examining Figs 3–5, it is evident that the depth of information flow from the center to the periphery of the communication networks varies significantly across the three types of Weibo content. The depth of flow typically refers to the measure of how deeply information or influence can travel through the network from a given node. It indicates the maximum reach of a node’s influence across the network’s layers or levels, highlighting how far information can propagate from the source node. The depth of flow in the opinion-based information network is particularly profound. Additionally, the node degree in these networks declines sharply as the layers of information flow increase. From an algorithmic perspective, a network’s flow depth is often analyzed using shortest path algorithms or breadth-first search (BFS) algorithms, especially when dealing with unweighted networks. In weighted networks, Dijkstra’s algorithm or variations might be used to compute the shortest paths, contributing to understanding the flow depth. In this context, "depth of flow" refers to the length of the Weibo information forwarding chain. It can show the path of Weibo information spread from the original post through various levels of sharing and engagement, providing insights into the virality, reach, and influence of the content on the network.

The other crucial metric is the "decay rate," which quantifies how quickly the influence or spread of information diminishes as it moves away from the source node across the network. It measures the rate at which the propagation strength or engagement level drops as the distance from the origin increases within the network layers. Algorithmically, the decay rate can be assessed by analyzing the reduction in node degree, edge weight, or another metric of influence as one moves to successive layers of the network from the source. In a social media context, for instance, this might translate to how the number of shares, comments, or likes decreases as the content moves further from the original poster in terms of network hops. The analysis of the decay rate is vital for several reasons:1. Understanding Information Lifespan: It helps to understand how long and far the information remains relevant or influential in the network. 2. Identifying Key Layers or Nodes: By observing where the decay rate changes significantly, researchers can identify potential layers or nodes that are crucial in sustaining or stifling the information spread. 3. Informing Dissemination Strategies: For example, interventions could be placed at points where the decay rate indicates a significant drop in information spread. Visually, the depth-of-flow characteristic is primarily reflected in the variation in the number of node layers, while the decay characteristic is primarily seen in the trend where the node size significantly decreases with the increase in distance from the starting node. Therefore, the researchers investigated the depth of flow along propagation paths and the rate of decay.

First, we calculated the network diameters and average path lengths of three distinct types of information communication networks. The results for the opinion-type information communication network were 11 and 1.553, respectively. For the information-type information communication network, the results were 9 and 1.180, respectively, and for the appeasement-type information communication network, the results were 10 and 1.175, respectively. Both the network diameter and the average path length of the opinion-type information communication network were the largest among the three types. This supports the observation that the depth of flow in the opinion-type information communication network is substantial, as evidenced by the data. Subsequently, we calculated the eccentricities of all nodes in the three types of information communication networks.

**Fig 7:**
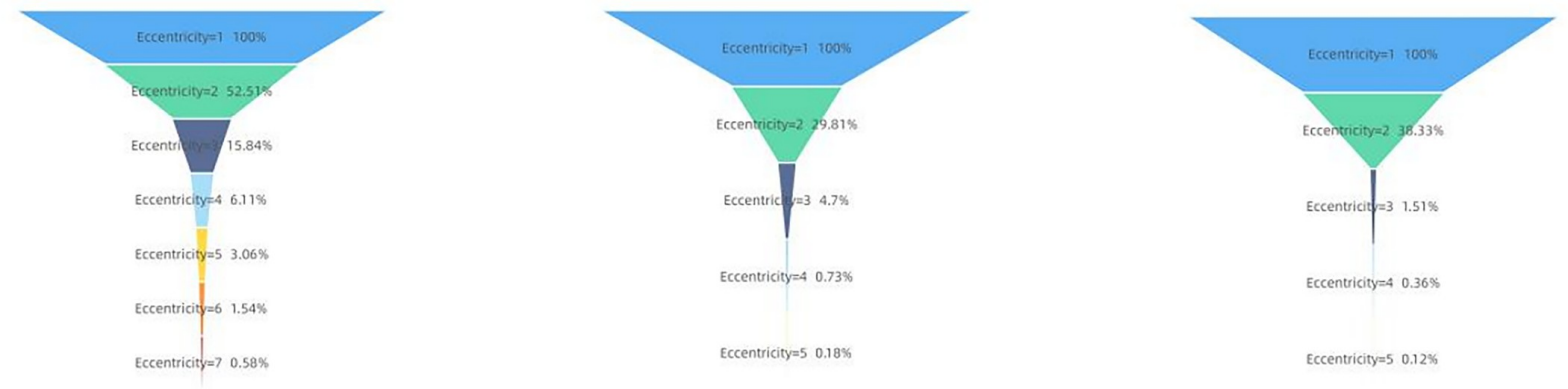
Ratios of decay with eccentricities of nodes.

In complex network analysis, "eccentricities" are defined as the calculations of the greatest distance (or maximum path) from a single node to all other nodes in the network. The eccentricity of a specific node signifies the maximum distance to other nodes. Within a network, the eccentricities of nodes assist in identifying the importance of a node in the network structure or its function in the spread of information. Nodes with smaller eccentricities suggest a closer connection to other nodes in the network, which could indicate a higher level of influence or more rapid capabilities for disseminating information. The ratios of their degrees’ decline as the eccentricities increase are depicted in Fig 7. The left, center, and right sections of the figure correspond to the opinion-based, information-based, and appeasement-based information communication networks, respectively.

A limited quantity of nodes aligns with high randomness and a reduction in generality, hence only the distributions of decay ratios with eccentricities exceeding 100 nodes are shown in Fig 7. As seen in Fig 7, the opinion-based Weibo content communication network exhibits a significantly lower average information decay rate compared with the other two types of communication networks, maintaining a certain node forwarding rate, even when faced with extended propagation paths. Notably, the information and appeasement-type communication networks once again demonstrate a significant similarity in their information decay rates.

The analysis of the provided data samples clearly distinguishes opinion-type, information-type, and appeasement-type Weibo content in terms of information communication networks, propagation nodes, propagation paths, and other factors. The specific conclusions are detailed below.

First, the communication networks for all three categories of Weibo content display an apparent scale-free characteristic. In this research, the "key minority" of nodes in the network significantly affects information communication. This property is particularly evident in the networks for information-type and appeasement-type communication. In appeasement-type posts, mainstream media’s agenda settings significantly affect public opinion’s direction. In information-type posts, authoritative news media have a substantial effect, indicating that the source of a report can influence its credibility and dissemination when the public is assessing the truth.

Second, within the communication networks for the three categories of Weibo content, the initiating nodes exert the most substantial influence on overall dissemination. However, the impact of these initiating nodes on overall dissemination is notably less potent in opinion-based posts compared to the other two categories. Conversely, the influence of the weaker nodes and the intermediary nodes on overall dissemination is more potent in opinion-based posts than in the other two types of information communication networks. This suggests that opinion-based information involves a higher degree of participation from all internet users, who demonstrate a stronger inclination to share and engage in the network communication of gender-related issues. These actions enable opinion-based posts to be propelled into a larger traffic pool by big data algorithms, increasing their visibility.

In network communication scenarios of comparable size, each level within a fan community collectively amplifies the spread of opinion-oriented posts. Unlike these posts, those that are information-centric or aimed at appeasement largely hinge on the diffusion impact of substantial, high-level nodes. This approach, also a typical method of information dissemination on Chinese social media, requires authoritative issuers and firsthand original data to augment its influence. The communication network for opinion-oriented data symbolizes the rising course of another grassroots discourse. It depends more on we-media and average internet users as the main force at key communication nodes rather than on official media, demonstrating a diffusion pattern of multilevel communication.

Moreover, networks for information-type and appeasement-type communication exhibit a notable similarity in node count and degree distribution. This indicates that, given the same propagation conditions, the dissemination of opinion-type information depends on the collective effort of all group levels. In contrast, information-type and appeasement-type information primarily rely on the influence of large, top-level nodes. In the network for opinion-type information communication, there needs to be more official media participants. The leading participating group comprises opinion leaders from various fields, social media influencers, and ordinary internet users. The communication network’s boundary is continuously expanded through propagation at each level.

Third, regarding transmission routes, the communication network for opinion-based Weibo content has the largest diameter, indicating that the depth of information flow from the network’s core to its periphery is the most extensive. More crucially, as previously noted, the count of female users significantly exceeds that of male users in all three types of Weibo posts, with the broadest gender disparity observed in opinion-based Weibo posts. This validates that women demonstrate a more pronounced emotional reaction and incentive to participate in online discourse within this event. In the framework of the public opinion brewing process on gender issues, the subjects tend to be contentious and polarizing. Users exhibit a heightened eagerness to engage in opinion-based Weibo debates because such debates allow users to articulate their views, stances, and outlooks, thereby establishing authentic public opinion or societal agreement that significantly affects the progression of real-world events. Moreover, the average decay rate of opinion-based Weibo information communication as the propagation path length increases is significantly lower than that of the other two types of information communication networks. This suggests that in online discussions about gender issues, audiences of opinion-based posts exhibit a greater level of active engagement and a stronger inclination to interact by commenting and sharing and to seek network alliances. Moreover, the information-based and appeasement-based information communication networks again show notable similarities in flow depth and decay rate.

## Conclusion

The primary contribution of this study is to provide a broad overview of gender issues in communication across Chinese social media. Analyzing three representative Weibo sample sets has examined the differences between the communication and interaction modes of various information types. The findings of this research explain why gender-related issues often incite controversy, opposition, and hate speech [80]. This is primarily because contentious discourse tends to engage opinion leaders and the public more effectively, and with the amplification provided by communication nodes of varying sizes, it exhibits a more potent cascading diffusion. In terms of internet governance, existing research indicates that moral judgments online are, to a degree, influenced by gender, and it is crucial for moderators of online discussions and platform providers to be aware of how gender stereotypes can affect these debates [40]. This study offers valuable insights into Internet governance strategies in China and globally. It can assist relevant government departments or internet platforms develop strategies for monitoring, filtering, and eliminating extreme rhetoric. Employing homophily mechanisms to assess the environment where radical speech arises, when there is a gender imbalance among participating users, serves as an indicator for potential gender-directed implications during the issue fermentation process.

## Discussion

This research has certain limitations. First, in selecting samples, we chose only three representative posts for network communication data collection without considering the timeline, thus failing to provide a comprehensive view of the evolution of public opinions. Second, the categorization into three types of information (information, opinion, and appeasement) does not sufficiently encompass all types of information present in "the 2022 Tangshan restaurant attack." This research represents only a slight advancement in the field. As feminist dialogues gain traction worldwide, both direct and indirect debates related to gender are increasingly prevalent on social networks. The trajectory of gender-related issues is intimately tied to local cultural norms, economic structures, and other factors. Traditional cultural research methods cannot provide timely feedback or handle large volumes of network data. Using computational communication research methods to examine online public opinions related to gender issues can address these shortcomings. This approach allows for a broader perspective and a better understanding of shifts in societal attitudes.
